# Stability of Major Geogenic Cations in Drinking Water—An Issue of Public Health Importance: A Danish Study, 1980–2017

**DOI:** 10.3390/ijerph15061212

**Published:** 2018-06-08

**Authors:** Kirstine Wodschow, Birgitte Hansen, Jörg Schullehner, Annette Kjær Ersbøll

**Affiliations:** 1 National Institute of Public Health, University of Southern Denmark, 1455 Copenhagen K, Denmark; ake@si-folkesundhed.dk; 2 Department of Groundwater and Quaternary Geological Mapping, Geological Survey of Denmark and Greenland, 8000 Aarhus C, Denmark; bgh@geus.dk; 3 National Centre for Register-based Research, Department of Economics and Business Economics, Aarhus BSS, Aarhus University, 8210 Aarhus V, Denmark; jsc@geus.dk

**Keywords:** drinking water, exposure assessment, sodium, potassium, magnesium, calcium, spatial variations, Denmark

## Abstract

Concentrations and spatial variations of the four cations Na, K, Mg and Ca are known to some extent for groundwater and to a lesser extent for drinking water. Using Denmark as case, the purpose of this study was to analyze the spatial and temporal variations in the major cations in drinking water. The results will contribute to a better exposure estimation in future studies of the association between cations and diseases. Spatial and temporal variations and the association with aquifer types, were analyzed with spatial scan statistics, linear regression and a multilevel mixed-effects linear regression model. About 65,000 water samples of each cation (1980–2017) were included in the study. Results of mean concentrations were 31.4 mg/L, 3.5 mg/L, 12.1 mg/L and 84.5 mg/L for 1980–2017 for Na, K, Mg and Ca, respectively. An expected west-east trend in concentrations were confirmed, mainly explained by variations in aquifer types. The trend in concentration was stable for about 31–45% of the public water supply areas. It is therefore recommended that the exposure estimate in future health related studies not only be based on a single mean value, but that temporal and spatial variations should also be included.

## 1. Introduction 

### 1.1. Geogenic Elements in Drinking Water

Drinking water based on groundwater resources contains geogenic elements which may be important long-term exposures to humans and may result in both harmful (e.g., arsenic) or beneficial (e.g., magnesium and calcium) health effects [1]. Concentrations of the elements in drinking water can be altered during water extraction and water treatment (e.g., aeration) at the waterworks, and the concentrations in drinking water are therefore not completely equal to concentrations found in groundwater.

### 1.2. Selected Cations

This study focusses on the four major cations in groundwater: sodium (Na), potassium (K), magnesium (Mg) and calcium (Ca) which are all geogenic and important for human health. 

The selected cations have all been related to cardiovascular diseases. For several decades, the positive association between high Na concentration in drinking water and increased risk of hypertension (HTN) has been studied [2]. In a recent study, it was argued that the association might be overstated and more complex than previously assumed [3], while the World Health Organization (WHO) concludes that the association is not conclusive [4]. Dietary K has been found to have a protective effect on systolic blood pressure in women, and the Na:K ratio in diet has been found to be associated with systolic blood pressure in both men and women [3].

In a systematic review of the association between cardiovascular disease and drinking water (including studies on Mg and Ca) in 2008, Catling et al. [5] found an association between increased Mg in water and a small reduction in cardiovascular mortality, but the evidence for an association between Ca and cardiovascular mortality was limited. 

A taste-related limit of 200 mg/L Na is recommended by WHO and, for that reason, the Danish drinking water quality specification is 175 mg/L [6]. WHO has not proposed guideline values for neither K, Ca nor Mg [1]. However, national limits have earlier been 10 mg/L for K, 50 mg/L for Mg and for Ca, the concentration should not exceed 200 mg/L [7].

Several studies have focused on defining baseline concentrations in groundwater (including major cations), e.g., for a chalk aquifer around Copenhagen [8] and in the United Kingdom [9], a coastal aquifer in South Africa [10] and a European project *“Natural baseline quality in European aquifers: a basis for aquifer management for the purpose of monitoring ground water quality”* [11]. Furthermore, the geochemistry of bottled water has been studied in e.g., Europe [12], Scandinavia [13] and Germany [14]. The typical ion concentration levels in drinking water are therefore known to some extent on a larger scale. In Denmark, concentrations of Na, K, Mg and Ca in the latest groundwater samples ranged from 5.6–10,000 mg/L, 0.45–120 mg/L, 0–855 mg/L and 2.5–820 mg/L, respectively [15].

### 1.3. Origin of the Four Major Cations in Drinking Water 

**Figure 1:**
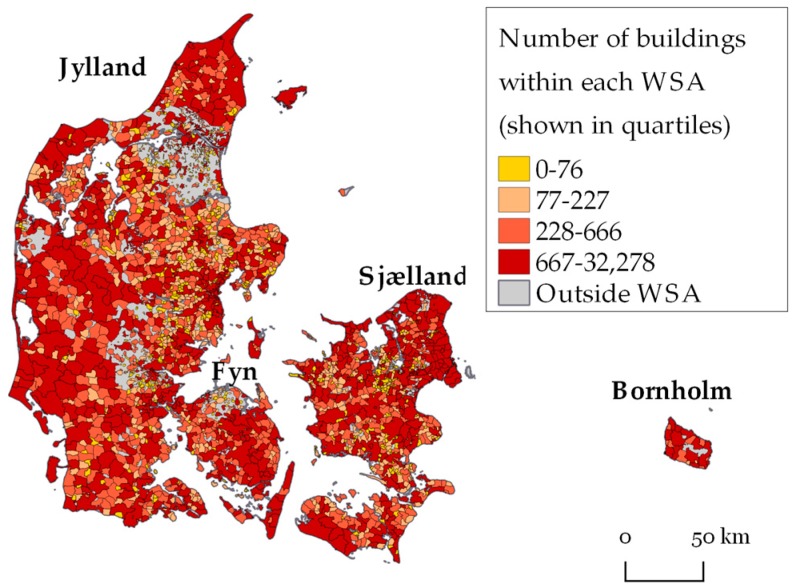
Public water supply areas (WSAs) (*N* = 2813 WSAs) in Denmark colored according to number of buildings within each WSA.

All tap water in Denmark is groundwater based [16] with the exception of a single small island (north-east of Bornholm, with around 80 consumers, see Figure 1 for a map of Denmark). 

The following is a list of the three most important aquifers in terms of extracted water volume: (1) Quaternary sand and gravel glacial deposits, (2) limestone and chalk from Upper Cretaceous and Danian, (3) and, quartz sand and micaceous sand from Upper Tertiary, with a distribution of approximately 58%, 24% and 10%, respectively [17]. Bornholm is the only place in Denmark where bedrock is present at the surface and used as an aquifer [17]. Since the western part of Denmark was not covered by ice during the latest Ice Age (Weichsel), the aquifers in this area have undergone a longer period of chemical weathering resulting in significant deeper acidification depth, and differences in the geochemical content of the groundwater, e.g., the content of Ca and Mg [17].

Na (atomic number 11) and K (atomic number 19) are alkali metals and occur in nature in the +1 oxidation state. In many rock-forming minerals, Na and K occur as important constituents [12], in Denmark mainly in feldspars $\left(\mathrm{Na},K,\mathrm{Ca},\mathrm{Ba}\right)\left(\mathrm{Al},\mathrm{Si}{)}_{4}O_{8}\right)$, micas (ex. biotite $K\left(\mathrm{Mg},\mathrm{Fe},\mathrm{Al}{)}_{2}{\mathrm{Si}}_{4}O_{10}{\left(\mathrm{OH}\right)}_{2}\right)$ and on cation exchange sites on clay minerals $\left({\mathrm{Al}}_{4}{\mathrm{Si}}_{4}O_{10}{\left(\mathrm{OH}\right)}_{8}O\right)$. Furthermore, Na occurs in halite (NaCl) or in porewater of old marine deposits. Groundwater recharge also contains Na due to e.g., atmospheric deposition, de-icing salt or artificial fertilizers [12,18]. 

Mg (atomic number 12) and Ca (atomic number 20) are chemically alkaline earth metals and occur in nature in a +2 oxidation state [12]. Mg and Ca are found in several minerals, where carbonates are the main mineral source in groundwater, since they are ubiquitous and have high dissolution kinetics [12]. In Denmark, the main sources are calcite $\left({\mathrm{CaCO}}_{3}\right)$ where Mg can substitute for Ca $\left({\mathrm{MgCO}}_{3}\right)$ [12,17], which are found both in small gravels in the Quaternary glacial deposits and as the major minerals in limestone. Furthermore, Ca and Mg as well as Na can be found on cation exchange sites on clay minerals, e.g., smectite [17].

### 1.4. Processes That Influence Concentration Levels

Chemical constituents are slowly infiltrated to or released in the aquifer, and the rate and amount of geochemical change is, among others, depending on groundwater recharge changes, anthropogenic input to the soil surface, atmospheric deposition, top soil type, water residence time and chemical weathering. In a Danish context, the three main processes in the aquifers regarding the four major cations are the carbonate system, solubility of minerals and cation exchange. 

The carbonate system is a buffer system against acid entering a carbonate rich aquifer and in relation to Ca, the governing equation is: ${\mathrm{CaCO}}_{3}+H^{+}↔{\mathrm{Ca}}^{2+}+{\mathrm{HCO}}_{3}^{-}$. The equilibrium is controlled by CO_2_, pH and the amount of available calcite. If the calcite buffer is depleted and acid (e.g., from oxidation processes) enters the aquifer, the pH can change, resulting in “aggressive water”, which is found in western Denmark [19]. 

Clay minerals and organic material have the largest cation exchange capacity (CEC) and cations are exchanged according to concentrations in the water and the affinity order: ${\mathrm{Al}}^{3+}>{\mathrm{Ca}}^{2+}>{\mathrm{Mg}}^{2+}>K^{+}>{\mathrm{Na}}^{+}>{\mathrm{Li}}^{+}$ [20]. Aquifers protected by thick clay layers, or layers with high levels of organic material, calcium carbonate or magnesium carbonate result in Na enriched water. 

These processes can be anthropogenically enhanced, unintentional and intentional. During water abstraction, there is a risk of water table drawdown resulting in increased pyrite oxidation and thereby increasing Ca and Mg concentrations due to production of acidification. Furthermore, over exploitation can lead to intrusion of recent sea water or deeper connate water, resulting in increased Na concentrations [8]. 

At the waterworks, concentrations can be changed during normal water treatment, e.g., where pH is controlled during treatment of acid water or in advanced water treatment as e.g., water softening. 

### 1.5. Drinking Water in Denmark and Exposure Assessment

The water supply structure in Denmark is mainly decentralized [16]. A total of 97% of the Danish population are supplied with tap water from about 2600 public water supplies and 3% are supplied with water from private smaller waterworks defined as wells serving less than 10 households [21]. Simple water treatment is sufficient at most waterworks; further, in 2012, only 74 waterworks had a permit for advanced water treatment of which only one was for water softening [22]. The drinking water quality is analyzed between 1/3 and 12 times per year, depending on the yearly amount of water abstraction [6]. Water samples are collected and analyzed by certified laboratories, that are required to report the analyses to the national database for groundwater and drinking water wells (Jupiter). The municipalities are the governmental supervisory authority and have to approve the analyses in Jupiter before they are publicly available [6]. Each waterworks supplies one to several administrative water supply areas (WSAs), and, furthermore, each WSA can be supplied by one to several waterworks. The decentralized water supply structure; close to 100% groundwater based drinking water; low consumption of bottle water [23]; and the relatively high number of drinking water samples registered in one national database are advantages in studies of drinking water and health, see e.g., [24,25,26,27,28,29]. 

### 1.6. Aim of the Study 

The concentration levels of Na, K, Mg and Ca in groundwater and drinking water in Denmark are known due to comprehensive monitoring, and collection in one single national database (Jupiter). When drinking water is used as exposure for diseases, it is important, especially for long term epidemiological studies, to know both the spatial and the temporal variations in exposure. However, the spatial variations and the stability of the concentrations across time in exclusively drinking water have not been studied before in a national Danish context. The aim of the present study is to examine the spatial and temporal changes in drinking water concentration of Na, K, Mg and Ca. The results will contribute with new knowledge for future studies in the field of environmental exposure assessment and impact on public health. Furthermore, the study contributes to the discussion on health effects of water softening, which has been requested lately by the consumers in Denmark [30], though possible health effects are not conclusive [31].

## 2. Materials and Methods

### 2.1. Study Design and Drinking Water Samples

Data on drinking water concentrations between 2 September 1892 and 29 June 2017 were extracted in end of June 2017 from the Jupiter database, administered by Geological Survey of Denmark and Greenland (GEUS). On waterworks-level, the database includes information on yearly water abstraction and water samples from both private and public waterworks [32] (See supplemental materials, Figure S1, for details on the data management workflow). 

Data were restricted to year 1980–2017, public waterworks and to drinking water samples after end treatment at the waterworks. A nationwide study on nitrate exposure from drinking water in Denmark was conducted in 2014 by Schullehner and Hansen [33], in which 2816 existing public WSAs were collected. In the present study, data have been updated to 2813 public WSAs.

In the Jupiter database, each drinking water sample is linked to a waterworks by a WaterworksID. For each WaterworksID a time series for each cation was created, and extremely low or high values (e.g., 0 mg/L and >200 mg/L difference in Ca concentration between the extreme value and the rest of the measurements at the waterworks) were manually validated. All analysis reports are stored in the Jupiter database. In case of extremely high or low concentrations these analysis reports were investigated for possible errors in decimal place. Furthermore, Na and K concentrations were compared with chloride (Cl) concentrations, and Mg and Ca concentrations where compared, since they are expected to be correlated. Extreme concentrations were excluded when no explanation was found for the measured concentration or when an error in decimal place was noticed.

Waterworks were linked to WSAs by spatial join in Quantum GIS version 2.18.14 (Quantum GIS, open source) [34]. When waterworks were located outside of the WSA, waterworks were manually linked based on information from the respective water supply companies and water supply plans from the municipalities [27]. Waterworks with no coordinates but with >10 drinking samples were manually matched to a WSA, by searching water supply plans and supply company webpages. Each waterworks distributes water to at least one WSA and each WSA can be supplied by several waterworks.

Difference in cation concentrations between water samples from waterworks and water samples from water pipes were compared in a scatterplot and tested with a multilevel mixed-effects linear regression model adjusted for date and waterworks were entered as a random effect. Due to a right skewed distribution of Na, K, and Mg, concentrations for these three cations were square-root transformed prior to the analyses. 

To compare drinking water concentrations of Na, Ca, K and Mg, WSAs were linked to their primary groundwater aquifer type. In 1996, GEUS initiated the development of a National Hydrological Model (DK-model) [35]. (For description of latest release of the model, see [36]). The geology is discretized in a 100 m × 100 m grid and the model consists of 16 hydro stratigraphic layers [37]. In the present study, the layers are simplified into the following four aquifer types: (1) Pre-quaternary hard rock (Hard rock) on the island of Bornholm, (2) Quaternary sandy deposits (Qs), (3) Tertiary sandy deposits (Ts), (4) and, Tertiary/Cretaceous limestone (Ls). The model contains lithological and hydrological information from wells in the national Jupiter database. An aquifer type was estimated in the model for 137,971 screened wells. After linking wells to waterworks based on ID number in the database, the primary aquifer was assigned to each waterworks. When more than one aquifer type was present, the primary aquifer was assigned as the one where ≥80% of the intakes were in, otherwise all respective aquifer types to the waterworks were listed. For WSAs supplied by >1 waterworks with different aquifer types, all aquifer types were kept.

### 2.2. Yearly Mean Concentration of the Major Cations

For each WSA and cation a yearly mean concentration was calculated. First, a yearly mean was calculated for each waterworks and year, for the period from oldest to youngest water sample for the given waterworks, starting in 1980. Then, for WSAs supplied by >1 waterworks, mean yearly concentration weighted by the yearly water extraction volume from each waterworks was calculated [24]. For waterworks supplying >1 WSA, the extracted water volume was divided by number of respective WSAs. 

### 2.3 Spatial Clustering of the Cation Concentrations: Na, K, Mg and Ca

For selected years (1980, 1990, 2000, 2010, 2015 and mean 2011–2015), areas with significant high or low concentrations were identified using yearly mean concentrations for each of the cations Na, K, Mg and Ca. Spatial scan statistics with a normal probability model was used to evaluate significance and approximate location of clusters [38]. An elliptic search window, 999 Monte Carlo replications and a maximum spatial cluster size of alternating 10%, 25% and 50% of the WSAs were applied in the analyses. The cluster analyses were based on centroids of WSAs. Due to a right skewed distribution of Na, K, and Mg, concentrations were square-root transformed prior to the cluster analyses. The cluster analyses were performed using the SaTScan™ software package v. 9.4.4.; (SaTScan™, Boston, MA, USA; https://www.satscan.org/).

### 2.4. Drinking Water and Aquifers

The association between type of aquifer and concentrations of Na, K, Mg and Ca was analyzed using a multilevel mixed-effects linear regression model. The association was adjusted for calendar year and WSA was included as a random effect. The analyses were followed by a pairwise comparison of the estimated marginal means to rank the aquifer types according to mean concentrations. Due to a skewed distribution of the concentration of Na, K, Mg and Ca, different transformations were considered including log, Box-Cox, square-root and rank transformation. Prior to the analyses, drinking water concentration for each cation was square-root transformed to obtain nearly normal distributed data. The analyses are limited to WSAs that are assigned with only one aquifer type. 

### 2.5. Temporal Trends in the Cation Concentrations: Na, K, Mg and Ca

To evaluate if the concentrations of Na, K, Mg and Ca were stable in the study period at WSA level, a temporal trend analysis was performed. Initially, scatterplots of concentration versus date of drinking water sample were created at WSA level for each cation. Each WSA was afterwards categorized into one of six trend categories (See supplemental materials, Figure S2, for further specifications of trend categories): 

*Too few*: Less than 3 drinking water samples.

*Constant:* Concentration interval < standard deviation of the concentration for all samples, excluding the 10% lowest and 10% highest measured concentrations. Furthermore, no significant increase or decrease in concentration during the study period.

*Significant increase/decrease:* Significant increase or decrease in concentration during the study period. Categorized based on linear regression where 95% confidence intervals of the trends were calculated and the null hypothesis (no trend over time) was significant (α = 0.05) and a degree of determination, *R*^2^ > 0.1

*Change-point:* Two or more different, but constant concentration levels or a significant change-point was observed (two connected regression lines). The change-point analysis was performed using two analyses. First, a Bayesian analysis with Markov chain Monte Carlo simulation of a change point regression model estimating the change point, and two slopes [39]. Secondly, the difference in concentration means before and after the change-point was tested with a *t*-test.

*Parallel:* Two or more constant concentration levels occurring at the same time were detected visually. Only WSAs which were not earlier categorized were included in the visual analysis.

*Fluctuating:* WSAs that do not fit into categories 1–5.

## 3. Results

### 3.1. Descriptive Results

**Figure 2:**
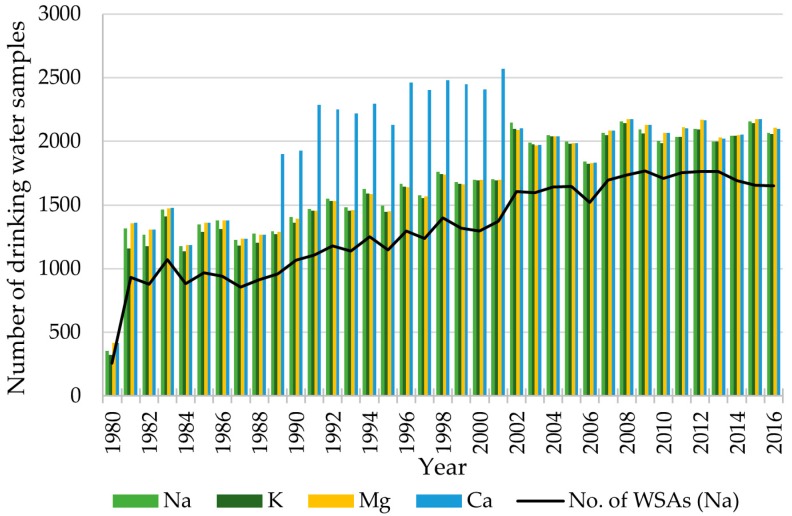
Number of drinking water samples per year for Na, K, Mg and Ca and number of water supply areas (WSAs) with water samples, 1980–2016.

The total number of drinking water samples per year (1980–2016) for Na, K, Mg and Ca increased from 1980 to 2002, from where on it was approximately stable until 2016, at a level of roughly 2000 drinking water samples per year per cation (Figure 2). Data for 2017 were only available for the first six months of 2017 and therefore omitted in the figure. For a period from 1988 to 2001, Ca was measured more frequently than the other cations, according to law [40,41]. Each year, the concentrations were measured at up to 1,900 different WSAs (Ca in 2001). Total number of WSAs was 2813.

The mean concentrations of the cations Na, K, Mg and Ca were 31.4 mg/L, 3.5 mg/L, 12.1 mg/L and 84.5 mg/L (Table 1). A total of 21, 30, 21 and 65 water samples were excluded as outliers for Na, K, Mg and Ca, respectively. Ca concentrations are normally distributed, whereas concentrations of Na, K and Mg are right skewed.

Waterworks have been linked for 2539, 2537, 2539, and 2549 WSAs with concentrations of Na, K, Mg, and Ca, respectively. A total of 99% of the waterworks have been assigned to a WSA. The 1% missing was due to lack of geographical coordinates of the waterworks.

### 3.2. Spatial Variations in Drinking Water Na, K, Mg and Ca

**Figure 3:**
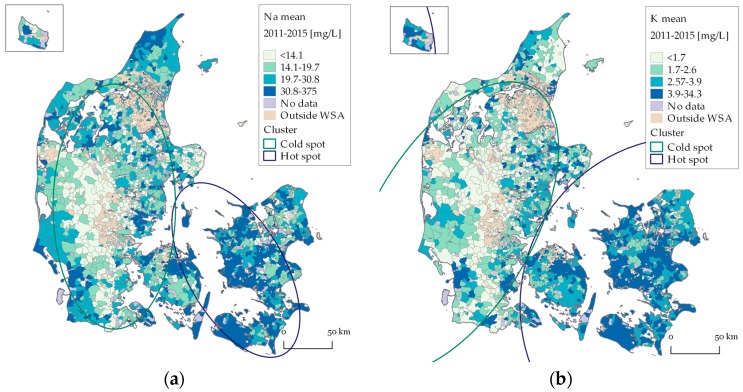

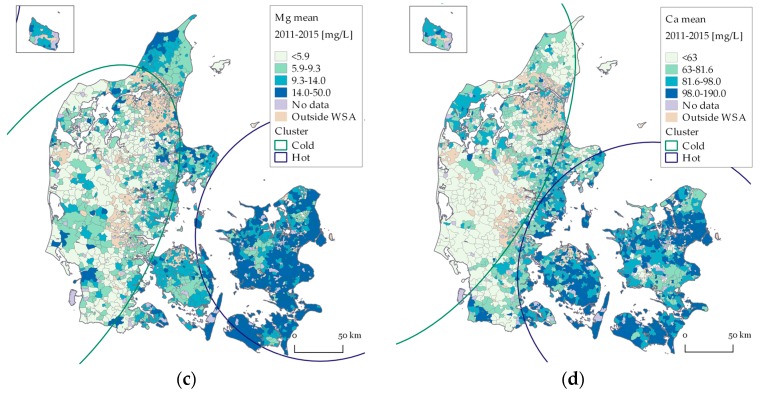
Spatial distribution of mean concentration of (**a**) Na; (**b**) K; (**c**) Mg; and (**d**) Ca, 2011–2015. Concentrations are shown in quartiles. In (**b**), Bornholm is included in the hot spot cluster.

Spatial variations in yearly mean Na, K, Mg and Ca concentrations in drinking water for each WSA (2011–2015) are shown in Figure 3a–d. Elliptic clusters of statistical (*p* ≤ 0.004) high or low concentrations overlaid on the maps are presented.

The geographical variation in all four cations are similar, with generally higher concentrations in the eastern part of Denmark (Sjælland and southern islands) and lower concentrations in the western part of Denmark. Furthermore, higher concentrations of Na and Mg are present in the northernmost part of Denmark and Na concentrations tend to be higher along the west coast of Denmark compared to the other cations. Notice the large concentration interval in the upper quartiles. For seven WSAs, the mean Na concentration is above the national recommended upper limit of 175 mg/L.

Number of WSAs included in the clusters, mean concentrations and *p*-values for cluster analyses of Na, K, Mg and Ca are presented in Table 2 (See supplemental materials, Table S1, for analyses results of transformed data). Change in number of included WSAs from 25% to 10% and 50%, respectively, in the cluster analyses did not change the overall results. Two significant clusters, one hot spot and one cold spot have been identified for all four cations, which enforced the earlier findings of an east–west pattern in concentrations. For Na, K and Mg the cold spot clusters are larger than the hot spot clusters, which underlines the earlier results of a right skewed distribution. Geographical extent of clusters for 1994, 2004 and 2014 resulted in similar east-west patterns (Figure 3a–d).

### 3.3. Aquifer Types Associated with Drinking Water Quality

**Figure 4:**
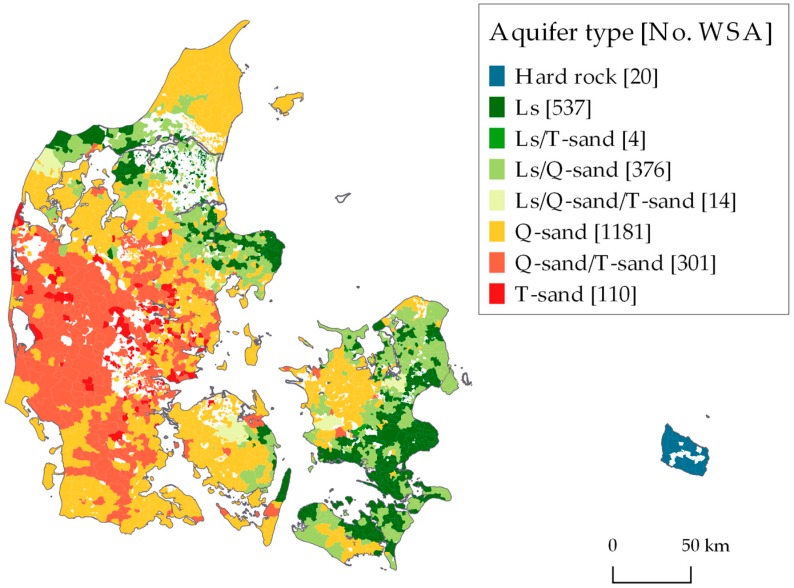
WSAs linked to main aquifer types in Denmark average 1980–2017. Ls = Tertiary/Cretaceous limestone, T-sand = Tertiary sand, Q-sand = Quaternary sand.

An aquifer type has been assigned for 2543 WSAs and the geographical range and number of WSAs for each aquifer type are presented in Figure 4. The results reflect the general geology in Denmark. Limestone and chalk dominates the eastern part of Sjælland, and the mid north of Jylland, whereas Quaternary sand and Tertiary sand dominates large parts of Fyn and Jylland. Bornholm is the only place in Denmark where the aquifer is in hard rock. For 696 WSAs, drinking water was extracted from more than one type of aquifer. 

The association between the four main aquifer types and drinking water concentrations of Na, K, Mg and Ca is shown in Table 3. For all four cations, the pairwise comparison of estimated marginal means showed that the mean concentration in Tertiary/Cretaceous limestone was higher than the mean concentration in Quaternary sand (*p*-value ≤ 0.001), which again was higher than the mean concentration in Tertiary sand (*p*-value ≤ 0.001). The mean concentration of the K cation in the Hard rock aquifer was significantly higher than in the three other aquifers. For Mg, the concentration in Tertiary/Cretaceous limestone and Hard rock aquifer was higher than in the two sand aquifers. For Ca, the mean concentration was significantly lower in the Tertiary sand aquifer compared to the mean concentration in the three other types of aquifers. For Na, the mean concentration in the Hard rock aquifer was not significantly different from the mean concentrations in any of the other aquifers (*p*-value > 0.1). Same overall results were obtained on log, square root and rank transformed data. 

### 3.4. Temporal Variations in Drinking Water cancentrations of Na, K, Mg and Ca 

**Figure 5:**
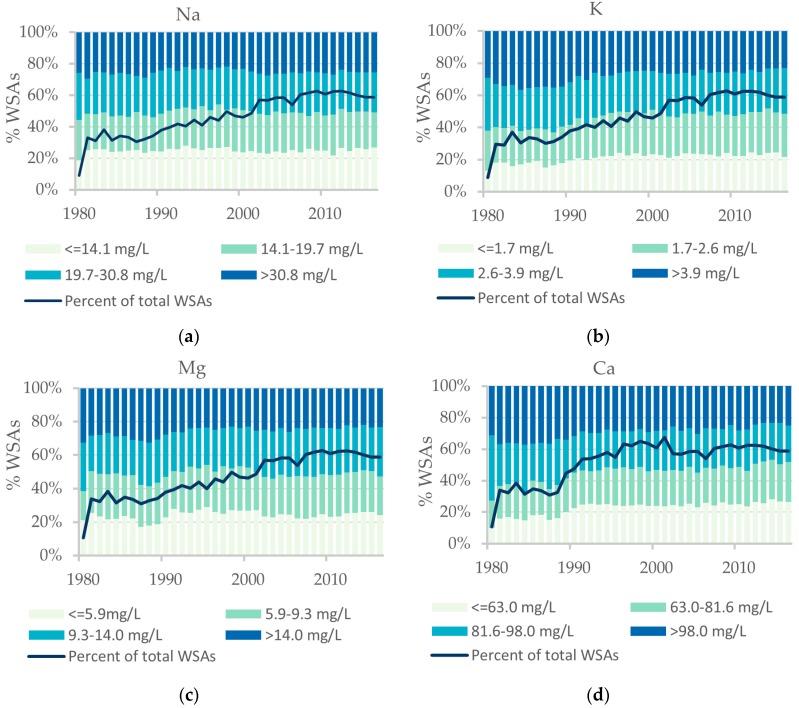
Temporal variations in yearly mean concentration at water supply area (WSA) level 1980–2016: (**a**) Na; (**b**) K; (**c**) Mg; (**d**) Ca. Concentration intervals are equal to quartiles of mean yearly concentration 2011–2015.

On a national scale the temporal variation in mean yearly concentration for all four cations is constant (1980–2016) (Figure 5). Percentage of total number of WSAs (2813) increases from the year 1980 to about 2002, from where on the yearly percentage of WSAs with samples is stable at about 60% of the total number of WSAs.

The result of the trend categorization shows that the number of WSAs in each category (*Too few*, *Constant*, *Decreasing/increasing*, *Change-point*, *Parallel* and *Fluctuating*) is similar for all four cations (Table 4). Between 25–41 WSAs have less than three samples, and were therefore categorized as *Too few*. The concentration trend is categorized as *Constant* for about 31% to 45% of WSAs. Twenty-two to 33% of the WSAs were categorized as *Decreasing/increasing* of which the change was more than ± the difference between the 25th and the 75th fractile for only 103, 79, 46, and 71 WSAs for Na, K, Mg and Ca, respectively (differences were 0.52 mg/L for Na, 0.07 mg/L for K, 0.27 mg/L for Mg and 1.07 mg/L for Ca). Just 2% to 4% of the WSAs are categorized as *Change-point* and 2% to 6% are categorized as *Parallel* trend. The second largest category is *Fluctuating* and 20% to 31% of the WSAs are in this group.

## 4. Discussion 

### 4.1. Key Findings 

The mean drinking water concentrations of Na, K, Mg and Ca for the period 1980–2017 are 31.4 mg/L, 3.5 mg/L, 12.1 mg/L and 84.5 mg/L, respectively. We found statistically significant geographical variations in all four cations, with higher concentrations in the east Denmark and lower in west. The pattern reflects the general geological structures in Denmark. We found that the concentrations were constant, or with only a little increase or decrease per year for about 60% of the WSAs. 

### 4.2. Comparison with Similar Studies

Median concentrations in comparison with concentrations found in selected studies are presented in Table 5. Our results are comparable with the median concentrations found in a similar sandy aquifer type in North Germany [42]. Compared to the concentrations in chalk in Denmark, both K, Mg and Ca are relatively lower, which underlines the finding of generally higher concentrations in chalk/limestone compared to Tertiary sandy aquifers. 

The geographical patterns in the drinking water concentrations of Na, K, Mg and Ca are similar to known concentration levels in groundwater, with higher concentrations in eastern Denmark and lower concentrations at the glacial outwash plain in the west. The results correspond to an earlier study on geogenic elements, where higher concentrations of iodine, lithium and strontium were found in eastern Denmark and lower concentrations in the west [46]. (High concentrations of iodine were, however, also found in the northernmost part of Denmark).

The slightly increased Na concentrations along the west coast of Jylland may be caused by high atmospheric deposition, flooding or saltwater intrusion in the aquifers. However, increased Na concentrations are not found specifically in coastal regions but also inland, where Na concentrations in groundwater are primarily caused by residual saltwater in marine deposits as e.g., chalk [8]. Furthermore, saltwater intrusion is expected to be most relevant in the distance of 200–300 meters from the coastline, and the size of the WSA might therefore weaken a possible relation. 

About 90% of the WSAs categorized as *Constant* trend are only supplied by one waterworks, whereas about 40% of the WSAs categorized as *Fluctuating*, are supplied by >1 waterworks (see Supplementary Data, Table S2). The variation in concentrations, are thereby firstly explained by changes in active extraction wells or change in the water supply structure, compared to natural changes in the aquifers. 

### 4.3. Limitations 

#### 4.3.1. Validity of Drinking Water Quality Data

During this study, misclassified concentration levels have been found and e.g., data on yearly extracted water volume are not complete. Lack of validation might influence the result, by blurring correct patterns and over or under estimation of concentrations. Compared to the high number of samples, this is expected to have a minor effect on the results.

The geographical variations of WSAs with assigned concentrations were analyzed, and the missing data were linked with specific geographical regions. Until around year 2000 there were no water samples from the former counties in southern Denmark, northern Denmark and west Sjælland. Water samples do exist before 2000, but no registration on sample purpose is listed in the database. Schullehner et al. [33] included nitrate samples not registered as drinking water samples. This would be incorrect in the present study where Ca can change during water treatment. The unequal geographical distribution of number and interval of water samples reduces the certainty of the temporal variations results and the distribution of trend categories. 

However, no association between the number of WSAs with water samples and the overall change in concentrations has been found, and the estimated trends are therefore assessed as a reliable indicator for the general trend in concentrations in drinking water. 

The trend category *Parallel* is partly subjectively estimated, and the estimated number of WSAs in the group is therefore an approximation. Statistically based criteria have been set up for the other categories, and it is expected that the overall tendency will not change. 

#### 4.3.2. Water Samples from Waterworks or Water Pipe 

Drinking water samples taken at the outlet of waterworks, in the water pipe system, and at the inlet to households were included in the study. Comparison of the groups for each cation showed no visual difference. The results from the multilevel mixed-effects linear regression model showed, that the mean concentrations were significantly different between the two groups for Na (*p*-value = 0.003) and Mg (*p*-value = 0.001). For both cations, the mean concentration was lowest in the water pipe system (5.4 mg/L for Na and 4.27 mg/L for Mg) compared to the waterworks, although only 146 and 148 measurements, respectively, were included in the analyses. For Mg, the difference was not statistically significant when the waterworks with highest concentrations (>40 mg/L) were excluded from the analysis, *p*-value = 0.132. Due to the low number of measurements and the fact that a small variation is expected because of time lag between the water samples at each waterworks, water samples from both waterworks and water pipes are included in the study.

#### 4.3.3. Public and Private Waterworks 

A total of 2444, 1249, 1284 and 1448 samples from private wells exist for Ca, Na, Mg and K, respectively. Concentration levels have visually been compared with concentration levels at nearest public WSA, and the concentrations were estimated to be similar. Schullehner et al. [33] found significantly higher concentrations of nitrate in private wells, which are mainly explained by higher agricultural nitrate pollution of the private wells compared to the public waterworks. 

#### 4.3.4. Classification of Groundwater Aquifers 

Classification of aquifers is based on a model, and uncertainties thereby exist. Although only WSAs assigned with one aquifer type are included in the regression analyses, the aquifer type is still an approximation to the aquifer type from which 80% of the water is extracted. The analyses have been tested on only WSAs categorized as a *Constant* trend. However, this resulted in too few observations in both Tertiary sand and Hard rock for the analyses to be valid. 

#### 4.3.5. Accuracy of Concentration Estimate

WSAs are assumed constant in time, which is a necessary simplification of reality. The misclassification of WSAs is expected to have a larger influence in areas with larger population growth compared to more rural areas. On the other hand, several of the well-fields to the WSAs for the capital are more than 30 years old. A larger change is found in the rural areas, where there is a tendency to change from private wells to public water supply. Since the geographical variations indicate that drinking water concentrations in a WSA are similar to neighboring WSAs the issue seems less important. 

### 4.4. Strengths 

Compared to other countries, the large number of water samples is an asset for the study. It results in a unique data set, for which the major cations otherwise might not be regularly measured and registered in a national database. The decentralized water supply structure, close to 100% groundwater based drinking water, low consumption of bottled water and the relatively high number of drinking water samples are advantages in studies of drinking water and health (see e.g., [24,25,26,27]). This has proven to be useful in the present study where the cation concentrations have not been studied at this detailed level earlier. 

Furthermore, the well-researched geology in Denmark is a strength for the study, improving the understanding of the mechanisms controlling the drinking water concentrations, and thereby revealing an opportunity for in future studies to define exposure variable concentrations based on the knowledge of geology and not only based on similar concentrations in the neighborhood. 

## 5. Conclusions

Yearly mean concentrations (2011–2015) of the major cations Na, K, Mg and Ca in drinking water in Denmark are estimated at 31.4 mg/L, 3.5 mg/L, 12.1 mg/L and 84.5 mg/L, respectively. A strong association has been found between aquifer types and drinking water concentrations, with decreasing concentration in the order of Tertiary/Cretaceous limestone, Quaternary sand and Tertiary sand. Our study shows that the concentrations of Na, K, Mg and Ca in drinking water are constant in time for about 31% to 45% WSAs. Where concentrations vary, the main explanation is that several waterworks are supplying the same WSA. In future studies, concentrations of Na, K, Mg and Ca in drinking water can be reasonably estimated as a yearly mean, despite lack of concentrations in space and time with a few exceptions. However, precaution must be applied when estimating correct exposure for drinking water consumers in about 23−36% of WSAs where concentrations have been identified as fluctuating or with two parallel concentration levels. 

## Figures and Tables

**Table 1:** Descriptive analysis of selected geogenic cations in drinking water in Denmark (1980–2017) given by means of number of drinking water samples, mean, median, 2.5% and 97.5% percentiles of the concentration for each of the cations Na, K, Mg and Ca.

Cation	No. of			Concentration (mg/L)			
	Samples	Waterworks	Samples Excluded	Mean	Median	2.5%	97.5%
Na	62,708	3724	20	31.4	20	9.1	130
K	61,581	3710	30	3.5	2.8	0.9	9.9
Mg	62,941	3724	21	12.1	9.8	2.8	35
Ca	72,561	3807	65	84.5	85	28.2	148

**Table 2:** Statistical significant clusters (*p* ≤ 0.05) of high (hot spot) and low concentrations (cold spot) of Na, K, Mg and Ca in drinking water. Up to 50% of the data points were included in the clusters, 2011–2015.

Cation	Type of Cluster	No. of WSAs		Mean Concentration (mg/L)		*p*-Value
		In Cluster	Total	Inside Cluster	Outside Cluster	
Na	Hot	500	2345	47.98	23.09	≤0.001
Na	Cold	1156	2345	20.74	35.83	0.002
K	Hot	902	2344	4.35	2.42	≤0.001
K	Cold	1154	2344	2.26	4.03	0.003
Mg	Hot	693	2345	18.19	8.14	≤0.001
Mg	Cold	1171	2345	7.02	15.19	≤0.001
Ca	Hot	1155	2344	96.24	66.48	≤0.001
Ca	Cold	894	2344	63.86	91.78	≤0.001

**Table 3:** Comparison of mean drinking water concentrations of Na, K, Mg and Ca between the four main aquifer types.

Type of Aquifers	No. WSAs ^2^	Concentration (mg/L)			
		Na	K	Mg	Ca
		Mean (*SD)*	Mean (*SD)*	Mean (*SD)*	Mean (*SD)*
Tertiary/Cretaceous limestone (1)	528	37.8 (38.0)	4.2 (3.6)	18.1 (10.5)	95.5 (28.4)
Quaternary sand (2)	1157	28.6 (27.2)	3.2 (2.5)	9.4 (5.0)	86.5 (27.2)
Tertiary sand (3)	110	17.5 (11.1)	2.2 (1.5)	6.7 (3.0)	63.8 (27.0)
Hard rock (4)	20	24.6 (15.1)	6.2 (5.2)	13.3 (5.5)	89.8 (21.4)
Order of aquifers ^1^	-	1 > 2 > 3	4 > 1 > 2 > 3	1 > 2 > 3 4 > 2 > 3	1 > 2 > 3 4 > 3

^1^ The order of aquifers is based on pairwise comparisons of estimated marginal means. An aquifer type is only listed where the difference in mean concentrations was significant (*p*-value ≤ 0.001); ^2^ Count of WSAs is only presented for Na.

**Table 4:** Categorization of drinking water concentrations of Na, K, Mg and Ca at water supply area (WSA) level according to the temporal distribution ranked in trend categories.

Cation	Total WSA	Too Few *n* (%)	Constant *n* (%)	Decreasing/Increasing *n* (%)	Change-Point *n* (%)	Parallel *n* (%)	Fluctuating *n* (%)
Na	2539	41 (1.6)	1136 (44.7)	723 (28.5)	56 (2.2)	86 (3.4)	497 (19.6)
K	2537	41 (1.6)	925 (36.5)	551 (21.7)	100 (3.9)	134 (5.3)	786 (31.0)
Mg	2539	42 (1.7)	1003 (39.5)	675 (26.6)	76 (3.0)	64 (2.5)	679 (26.7)
Ca	2549	25 (1.0)	786 (30.8)	831 (32.6)	103 (4.0)	148 (5.8)	656 (25.7)

**Table 5:** Median concentration (mg/L) of the four cations in the present study in comparison to other studies.

	Concentration (mg/L)						
Cation	Present Study	European Tap Water [43] ^1^	European Bottled Water [43] ^1^	Copenhagen Baseline (Chalk) [8]	North Germany Groundwater [42]	UK Chalk [44]	Groundwater Slovakia [45] ^2^
Na	20.0	9.47	17.8	19	19.3	36	20.34
K	2.8	1.6	2.5	4	3.4	6.8	11.10
Mg	9.8	9.61	18.9	19	9.1	19	28.29
Ca	85.0	59.5	76.3	114	71	57	93.56

^1^ Euro Geo Surveys (EGS) 2010, in [43]; ^2^ Concentration given as mean value.

## Supplementary Materials

The following are available online at http://www.mdpi.com/1660-4601/15/6/1212/s1, Figure S1: Workflow, Figure S2: Description and selected illustrations of the different trends in concentration, Table S1: Statistical significant clusters, Table S2: Comparison between number of waterworks and trend in concentration.
