# Supplementary material for: Stability of Major Geogenic Cations in Drinking Water—An Issue of Public Health Importance: A Danish Study, 1980–2017
Source: Int J Environ Res Public Health. 2018 Jun 8;15(6):1212. doi: 10.3390/ijerph15061212 (PMC6024924; doi:10.3390/ijerph15061212)
Supplement: Supplementary file 1 [file ijerph-15-01212-s001.pdf]

Supplementary Materials

Figure S1: Workflow

Workflow describing the data management for combining data sets into new data sets and how these are used in statistical analyses.

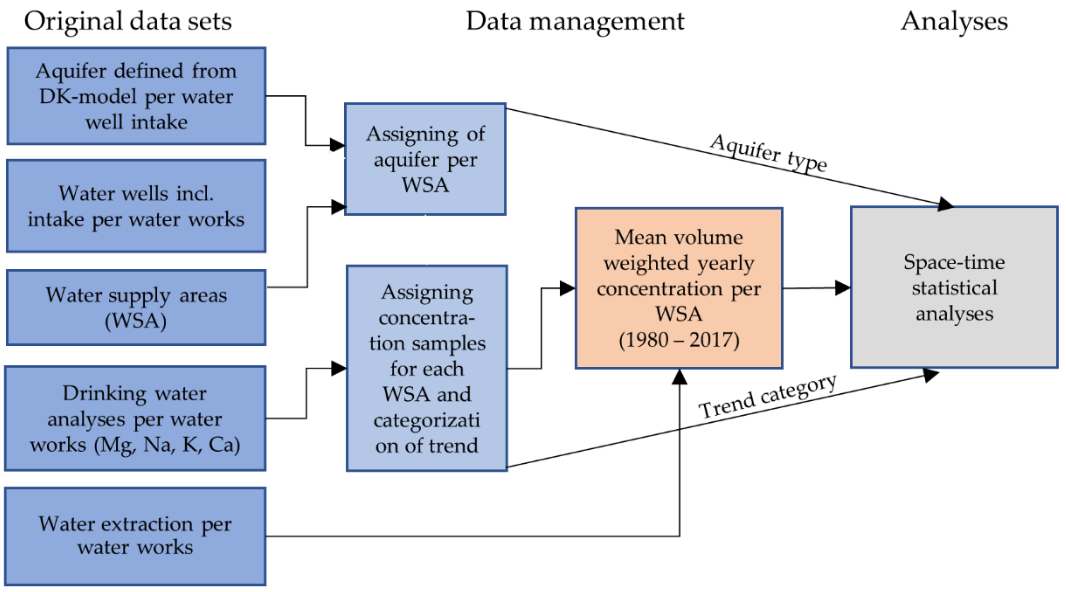

Figure S1: Workflow during data management and analyses. Stippled lines indicate that data sets are used in the discussion of the results as well as in the analyses.

Figure S2: Additional description of trend categories.

Description	Illustration
Constant: Concentration interval < standard deviation of the concentration for all samples, excluding the 10% lowest and 10% highest measured concentrations. Furthermore, no significant increase or decrease in concentration during the study period.	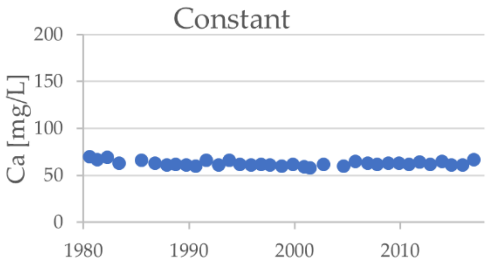
Significant increase/decrease: Significant increase or decrease in concentration during the study period. Categorized based on linear regression where 95%-confidence intervals of the trends were calculated and the null hypothesis (no trend over time) was tested on a significance (α=0.05) and a degree of determination, $R^2 > 0.1$	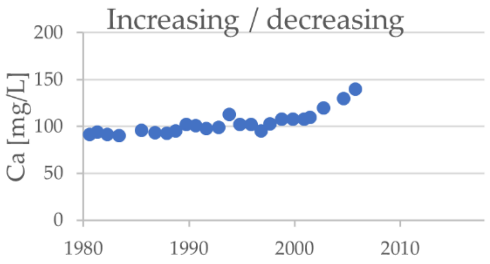
Change-point: Two or more different, but constant concentrations level or a significant change-point was observed (two connected regression lines). The change-point analysis was performed using two analyses. First, a Bayesian analysis with Markov chain Monte Carlo simulation of a change point regression model estimating the change point, and two slopes [39]. Secondly, the difference in concentration means before and after the change-point was tested with a t-test.	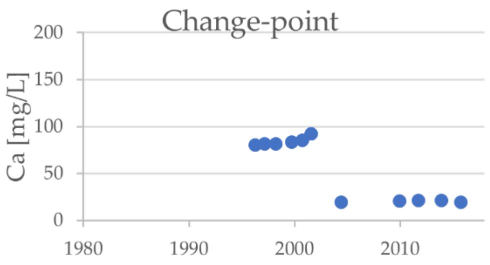
Parallel: Two or more constant concentration levels occurring at the same time were detected visually. Only WSAs which were not earlier categorized were included in the visual analyze.	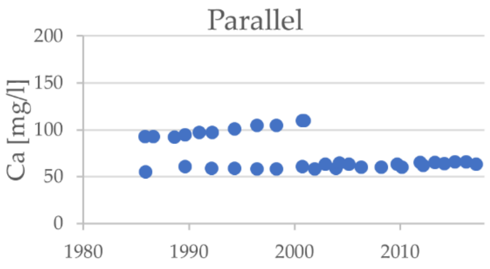
Fluctuating: WSAs that do not fit into categories 1-5.	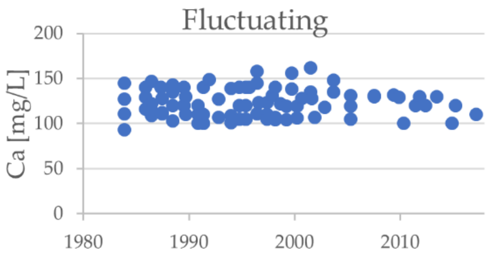

Figure S2 Description and selected illustrations of the different trends in concentration. All examples are for Mg.

Table S1: Additional cluster analyses

Table S1 Statistical significant clusters (≤ 0.05) of high (hot spot) and low concentrations (cold spot) of Na, K, Mg and Ca in drinking water. Up to 50% of data points were included in the clusters. All clusters are of mean concentration 2011-2015.

**Cation**	**Transformation**	**Type of cluster**	**No. of WSA**		**Mean conc. [mg/L]**		***p*-value**
			In cluster	Total	Inside cluster	Outside cluster	
Na	None	Hot	500	2345	47.98	23.09	≤0.001
Na	None	Cold	1156	2345	20.74	35.83	0.002
Na	Log	Hot	537	2345	36.23	19.30	≤0.001
Na	Log	Cold	980	2345	17.12	26.84	≤0.001
Na	Square root	Hot	537	2345	41.22	20.88	≤0.001
Na	Square root	Cold	1073	2345	18.75	30.80	≤0.001
K	None	Hot	902	2344	4.35	2.42	≤0.001
K	None	Cold	1154	2344	2.26	4.03	0.003
K	Log	Hot	999	2344	3.74	2.01	≤0.001
K	Log	Cold	1172	2344	1.97	3.53	≤0.001
K	Square root	Hot	914	2344	4.08	2.22	≤0.001
K	Square root	Cold	1048	2344	2.04	3.65	≤0.001
Mg	None	Hot	693	2345	18.19	8.14	≤0.001
Mg	None	Cold	1171	2345	7.02	15.19	≤0.001
Mg	Log	Hot	899	2345	15.18	6.75	≤0.001
Mg	Log	Cold	1154	2345	6.17	13.46	≤0.001
Mg	Square root	Hot	693	2345	17.39	7.67	≤0.001
Mg	Square root	Cold	1170	2345	6.60	14.36	≤0.001
Ca	None	Hot	1154	2344	95.92	66.14	≤0.001
Ca	None	Cold	894	2344	63.55	91.44	≤0.001

Table S2: Comparison of number of waterworks and concentration trend

Table S2 Comparison between number of waterworks and trend in concentration. Data is presented for Ca.

Trend in concentration	One waterworks per WSA	More than one waterworks per WSA
Too Few	25	0
Constant	719	67
Decreasing/increasing	680	151
Change-point	81	22
Parallel	9	139
Fluctuating	380	277
